# Biogenic ZnO Nanoparticles Synthesized from *Origanum vulgare* Abrogates Quorum Sensing and Biofilm Formation in Opportunistic Pathogen *Chromobacterium violaceum*

**DOI:** 10.3390/pharmaceutics13111743

**Published:** 2021-10-20

**Authors:** Majid Rasool Kamli, Maqsood Ahmad Malik, Vartika Srivastava, Jamal S. M. Sabir, Ehab H. Mattar, Aijaz Ahmad

**Affiliations:** 1 Department of Biological Sciences, Faculty of Science, King Abdulaziz University, Jeddah 21589, Saudi Arabia; jsabir2622@gmail.com (J.S.M.S.); emattar@kau.edu.sa (E.H.M.); 2 Center of Excellence in Bionanoscience Research, King Abdulaziz University, Jeddah 21589, Saudi Arabia; 3 Department of Chemistry, Faculty of Science, King Abdulaziz University, Jeddah 21589, Saudi Arabia; 4 Clinical Microbiology and Infectious Diseases, School of Pathology, Faculty of Health Sciences, University of the Witwatersrand, Johannesburg 2193, South Africa; vartika.srivastava@wits.ac.za (V.S.); Aijaz.Ahmad@wits.ac.za (A.A.); 5 Infection Control Unit, Charlotte Maxeke Johannesburg Academic Hospital, National Health Laboratory Service, Johannesburg 2193, South Africa

**Keywords:** ZnO nanoparticles, biofilm, quorum sensing, *Chromobacterium violaceum*

## Abstract

This study presents an inexpensive, eco-friendly, and simple green synthesis of ZnO nanoparticles using *Origanum vulgare* extract. These nanoparticles are non-hazardous, environmentally friendly, and cheaper than other methods of biosynthesis. Ongoing research determines the role of phytochemicals in the fabrication and biosynthesis of ZnO NPs and their role in antibacterial activity and biomedical applications. Characterizations by fourier transform infrared spectroscopy (FTIR), diffuse reflectance UV-visible spectroscopy, X-ray diffraction (XRD), scanning electron microscopy (SEM), and transmission electron microscopy (TEM) determine the successful biosynthesis of ZnO NPs. Meanwhile, TEM and X-ray diffraction studies approximated the spherical morphology and crystalline nature of biosynthesized ZnO NPs of nano size in the range of 20–30 nm. The global increase in drug resistance necessitates the search for new drugs with different mechanisms of action. Quorum sensing (QS), a cell-to-cell communication, has gained attention as an emerging drug target. It controls numerous biochemical processes in bacteria, which are essential for their survival and pathogenicity. The potential of nanomedicines has also been tested to synthesize new antibiotics to tackle drug resistance. ZnO NPs were explored for their antibacterial, antiquorum sensing, and antibiofilm activities with a bioreporter strain of *Chromobacterium violaceum*. Susceptibility testing results indicated the potential antibacterial activity of ZnO NPs with a minimum inhibitory concentration (MIC) of 4 µg/mL and minimum bactericidal concentration (MBC) of 16 µg/mL. Antiquorum-sensing assays revealed that these nanoparticles inhibit quorum sensing with minimum antiquorum sensing activity (MQSIC) of 1 µg/mL, without causing any bacterial growth inhibition. In addition, ZnO NPs inhibit biofilm formation at inhibitory and higher concentrations. RT-qPCR results supported the downregulation of the quorum sensing genes when *C. violaceum* was treated with ZnO NPs. The outcomes of this study are promising with regard to the biofilm and quorum sensing, emphasizing the potential applications of ZnO NPs against bacterial communication and biofilm formation.

## 1. Introduction

Nowadays, extensive research is being carried out in nanotechnology owing to various scientific and technological fields, including pharmacology, mechanical industry, drug delivery etc. [1,2,3]. Interest in the use of nanomaterials has increased exponentially because of the smaller size, large surface-to-volume ratio, and tunable morphological properties [4]. Biosynthesized nanomaterials have recently shown proven worth applicable in various fields, including biomedical science, health, magnetic science, chemical industries, electronics, space industries, mechanics, drug and gene delivery, and have gained immense attention [5]. However, biosynthetically prepared NPs are more economical, robust, and environmentally friendly than a synthesis techniques [6]. The ZnO has emerged as a potential candidate to be used in industry as it is nontoxic, has greater photoactivity, is inexpensive, antimicrobial, and biocompatible [7,8,9,10,11], and has innumerable applications in the fields of cosmetics, food preservation, nanomedicines and electrode, biosensor, photocatalyst, and solar cell manufacturing [11,12]. Crystalline morphology and surface area ZnO NPs are used in bioactive applications, such as biosensing, antimicrobial preparations, catalysis, photonics, optoelectronics, and other technological applications [13,14]. The synthesis techniques also determine the ZnO properties. Many methods are used to fabricate ZnO NPs, such as thermal decomposition, spray pyrolysis, chemical precipitation, hydrothermal, and sol and gel methods [15,16,17]. However, the eco-friendly, more economical, and quickly adopted procedure of green synthesis has proven more advanced in the production of metal oxide-NPs [18]. ZnO biosynthesis provides more eco-friendly material with efficient photocatalytic activity, well-defined crystal structure, and high purity. The literature survey revealed that different metal oxide NPs like Zn, Ag, Mg, Cu, Al, etc. [19,20] were synthesized through the biogenic model for other economic applications. The ZnO has been biosynthesized from various plants like *Abutilon indicum* [21], *Maringa oleifera* [22], *Cinnamomum verum* [23], *Ixora coccinea* [24], and *Punica granatum* [25], which have shown excellent photocatalytic activities [26]. Origanum vulgare is a perennial herb belonging to the family Lamiaceae and has an essential role in phytotherapeutic applications. Carvacrol and thymol are the two main components of *Origanum vulgare* oil, which has antibacterial properties [27,28] and is used as a food flavoring with a high content of phenolic derivatives [29]. It has been reported that *Origanum vulgare* extract exhibited antimicrobial properties against some strains of Gram-positive and Gram-negative bacterial strains, such as *P. aeruginosa*, *Bordetella bronchiseptica*, *Escherichia coli*, *Burkholderia cenocepacia*, *Acinetobacter lwoffii*, *Bacillus subtilis*, and *S. aureus* [30]. Further, essential oil obtained from *Origanum vulgare* inhibits the biofilm formation of *P. fluorescens* strains and alters their motility [31]. 

The discovery of new antibiotics has been believed to be one of the most outstanding accomplishments of the last century. Since the discovery of penicillin, hundreds of antibiotics have been introduced in clinics to treat different bacterial infections. Ultimately, these developed antibiotics are designed with many mechanisms for the drug action and are fundamental for killing or inhibiting bacterial growth. Unfortunately, these traditional mechanisms pose survival pressure on the bacteria, and promote their resistance to these antibiotics [32,33,34]. As a result, antibiotic resistance is a common challenge in clinics treating different bacterial infections. The other important factor amplifying drug resistance is biofilm formation in bacterial pathogens, which are the most challenging factors to treat [35]. Therefore, besides the need to find new antibiotics, there is a pressing demand for novel strategies to target the pathogens’ viability.

*Chromobacterium violaceum* is mainly found in tropical and subtropical regions and is abundant in soil and water. This microbe has been well explored for violacein production and is considered a bioreporter for identifying anti-quorum sensing molecules. Several reports mention the biotechnological and therapeutic relevance of *C. violaceum*, predominantly the purple pigment, violacein [36,37,38]. Human infections are generally linked to high mortality rates, life-threatening sepsis, and germs spreading quickly to multiple organs, including the liver, lungs, and spleen. Exposure of wounds and traumatic lesions to soil and water carrying *C. violaceum* is a typical route of transmission [39]. In addition, the increasing problem of drug resistance among microbes and the high dissemination rate of drug-resistant pathogens are the biggest concerns and challenges for the therapeutic sector [40]. Although biofilm formation is mainly used as a mechanism for enhancing drug resistance in bacterial species, it acts as a barrier against the external therapeutic environment and cells embedded into the biofilm, leading to treatment failure. It has been widely reported that biofilm formation is a highly regulated process and involves bacterial communication, known as quorum sensing (QS), which controls various physiological functions of bacteria, including pathogenicity [41]. Therefore, inhibition of QS will inhibit biofilm-forming bacteria’s growth and survival and reduce drug resistance among pathogens.

So far, several natural and synthetic compounds have proved to have anti-quorum sensing potential; however, due to several other factors, none of them have proceeded to the higher stages of drug development [42,43]. Nanomaterial-based therapies, which can evade existing mechanisms associated with acquired drug resistance, are promising tools for combating difficult-to-treat bacterial infections [44]. Metal and metal oxide NPs are promising new options for fighting antibiotic resistance in Gram-negative bacteria by acting as QS inhibitors [45]. It has been reported that selenium and tellurium-based NPs disrupt quorum sensing and the formation of biofilm in *C. violaceum* [46]. Therefore, the production of semi-synthetic analogs or nanoparticles derived from natural sources with specific properties is of interest in developing anti-quorum sensing agents [47]. 

We focused our research on biosynthesis, being non-toxic and non-polluting, as a simple procedure for developing ZnO NPs of *Origanum vulgare* leaf extract. Simultaneously, these bio-synthesized ZnO NPs were used as anti-quorum-sensing and anti-biofilm agents against a bio-reporter strain of *C. violaceum*. 

## 2. Materials and Methods

### 2.1. Chemicals

The reagents, such as zinc nitrate hexahydrate (Zn(NO_3_)·6H_2_O) (98%) and sodium hydroxide (NaOH) (98%), acquired from Sigma-Aldrich (St. Louis, MO, USA), were used without further purification. The *Origanum vulgare* leaves were purchased from the local supermarket in Jeddah, Saudi Arabia. Double-distilled water was used as the synthesis medium.

### 2.2. Preparation of Aqueous Leaf Extract of Origanum vulgare

To prepare *Origanum vulgare* extract, freshly collected leaves of *Origanum vulgare* were thoroughly washed and dried in air for about 2–3 days. The dried leaves were ground to form a fine powder. The powder obtained was then immersed in 100 mL of distilled water in a 250-mL Erlenmeyer flask. The mixture was then heated at 70 °C for one hour and cooled down to be filtered through Whatman number-1 filter paper. The collected filtrate was stored at 4 °C for further use in preparation of ZnO NPs. 

### 2.3. Synthesis of ZnO NPs Using Origanum vulgare Extract

The ZnO NPs were prepared by a typical procedure in which 50 mL of the preserved *Origanum vulgare* extract were added to 2 M zinc nitrate solution in a 250-mL Erlenmeyer flask. The whole mixture was then heated under continuous stirring at 60 °C for 2 h on a magnetic hotplate stirrer. The continuous stirring of the reaction mixture helps in the electrostatic interaction between the Zn^2+^ ions and the biomolecules of *Origanum vulgare* leaf extract, which acts as a stabilizing and reducing agent [48]. Nanoparticle formation and stabilization take place through the nucleation, shaping, and growth process. The synthesis of ZnO NPs can be monitored by visual observation during which a clear illustration of change in color of the reaction mixtures takes place due to formation of ZnO NPs [49]. The color of the solution, which turned from light yellow to a darkish yellow color, precipitate confirms the formation of ZnO NPs [50]. This colored reaction mixture was centrifuged by Thermo scientific IEC (CL31 Multispeed Centrifuge) at 10,000 rpm for 20 min to collect the as prepared ZnO NPs, washed several times with ethanol, and deionized waster to get rid of impurities. Subsequently, the solid material was placed in a silica crucible maintained at 150 °C using an electric Bunsen burner for about 30 min. The ZnO nanoparticles obtained were then poured into 50 mL of double-distilled deionized water followed by centrifugation at 4000 rpm for 10 min, and this process was repeated two times and finally dried at 100 °C. The collected ZnO NPs were further calcined at 400 °C for 2 h, and the acquired powder was used for further characterization and analysis. 

### 2.4. Characterization of Biogenic ZnO NP

In the wavelength range of 200–800 nm, UV-Visible diffuse reflectance spectra were obtained using UV-Vis DRS, Shimadzu 2450. A JEOL TEM (Hitachi, Ltd., Tokyo, Japan) was used to examine the shape and particle size of the ZnO NPs. A 5-μL drop of ZnO NPs suspension was placed on a carbon-coated copper grid for TEM investigation. The excess liquid was collected with filter paper after 2 min. The sample was permitted to dry in the open air. X-ray diffraction (XRD) patterns were measured in the 2θ values range from 20 to 80, with a scanning rate of 2°/minute using an X-ray diffractometer (model XPERT-PRO) operating at 40 KV and 30 mA (Cu K radiation (k = 1.5406)). FTIR (Bruker) was used to determine the related functional groups on ZnO NPs and cap and reduce agents inside the extract. Scanning electron microscopy (SEM) was used to study the surface morphology of ZnO NPs, and energy-dispersive X-ray spectroscopy (EDX) was used to determine the chemical composition of the synthesized ZnO NPs. The weight loss of the ZnO NPs as a function of temperature was measured using a Q500 instrument (TA Instruments) in the temperature range of 30 to 800 °C. The hydrodynamic diameter and zeta potential were measured in an aqueous medium with dynamic light scattering (Zetasizer nanoseries, Malvern, UK) to determine the size and stability of ZnO NPs.

### 2.5. Bacterial Strain and Growth Conditions

In this study, a bioreporter strain of *C. violaceum* ATCC12472 was used. The strain was obtained from ATCC and stored at −80 °C supplemented with 20% glycerol, in the Department of Clinical Microbiology and Infectious Diseases, University of the Witwatersrand, Johannesburg, South Africa. Before experiments, the *C. violaceum* ATCC12472 was revived by culturing on Luria–Bertani (LB; Sigma Aldrich Co., Saint Louis, MO, USA) agar plates at 30 °C for 24 h.

### 2.6. Antimicrobial and Antiquorum-Sensing Testing

To study the antibacterial and antiquorum-sensing activities of ZnO NPs, susceptibility assays including determination of the minimum inhibitory concentration (MIC), minimum bactericidal concentration (MBC), and minimum antiquorum-sensing concentration (MQSIC). These were determined by micro broth dilution assay following the Clinical and Laboratory Standards Institute (CLSI) guidelines of reference document M07-A10. A stock solution (100 µg/mL) of biogenic ZnO NPs was prepared in 1% dimethyl sulfoxide (DMSO; Sigma Aldrich Co., USA). The MIC was the lowest concentration of the biogenic ZnO NPs and resulted in visible growth inhibition. Furthermore, the MBC value for the ZnO NPs was assessed by sub-culturing 10 µL from wells showing no growth on LB plates. Results were recorded after incubating the plates at 37 °C for 24 h. For each experiment, positive (ampicillin), negative (1% DMSO), sterility (cell free), and growth (compound free) controls were included. At the same time, the well with turbid growth but no color was considered as MQSIC of the biogenic ZnO NPs.

### 2.7. Antiquorum-Sensing Activity

The anti-quorum-sensing activity of biogenic ZnO NPs was assessed both qualitatively and quantitatively. 

### 2.8. Qualitative Antiquorum-Sensing Activity

The qualitative antiquorum activity of biogenic ZnO NPs was assessed following the agar plate method as described previously [34]. Briefly, 0.5 MacFarland stock solution of *C. violaceum* ATCC12472 was seeded on an LB agar plate, and filter discs were infused with 20 µL of biogenic ZnO NPs at MQSIC sub-MQSIC were placed on it. Thereafter, plates were kept at 30 °C for 24 h, followed by observation of clear zones, indicating that growth and quorum sensing were inhibited, and opaque zones, indicating the inhibition of quorum sensing. All the zones were measured in millimeters (mm) against a violet background of bacterial growth. In every set of experiments, a negative control (1% DMSO) was included. 

### 2.9. Quantitative Antiquorum-Sensing Activity

The antiquorum-sensing property of ZnO NPs was quantified by calculating the inhibition of violacein pigment in treated *C. violaceum* cells and comparing it with untreated cells using the protocol described previously [34]. Briefly, a single pure colony of *C. violaceum* ATCC12472 was propagated in LB broth (5 mL) alone (untreated control) and supplemented with MQSIC and sub-MQSIC (0.25 and 0.5 MQSIC) of biogenic ZnO NPs. The cultures were then incubated for 24 h with shaking at 30 °C. An aliquot of 1 mL from each tube was centrifuged at 12,000× *g* for 10 min, and pellets were dissolved in 1 mL of 100% DMSO. Different controls included in this test were eugenol (positive control), 1% DMSO (negative control), and growth control. Eugenol was used as a positive control instead of a known antibiotic as there are no known antibiotics with antiquorum-sensing capabilities. In contrast, eugenol has been reported to have antiquorum-sensing activity [51]. The violacein quantification at OD_585nm_ and the percentage inhibition of violacein was calculated as follows:

(1) $$\mathrm{Percentage}\;\mathrm{of}\;\mathrm{violacein}\;\mathrm{inhibition}=\frac{\mathrm{control}\;{\mathrm{OD}}_{585\mathrm{nm}}-\mathrm{test}\;{\mathrm{OD}}_{585\mathrm{nm}}}{\mathrm{control}\;{\mathrm{OD}}_{585\mathrm{nm}}}\times 100$$

The quantity of violacein produced in the negative control was used to determine MQSIC of ZnO NPs. It was considered the minimum concentration responsible for inhibiting violacein production in *C. violaceum* ATCC12472 ≥ 50%.

### 2.10. Biofilm Inhibition Assay

To establish the anti-biofilm property of ZnO NPs, the crystal violet method using a 96-well flat-bottomed microtiter plate was employed [52]. *C. violaceum* cells were cultured in LB broth, supplemented with and without different concentrations of the biogenic ZnO NPs (0.5 × MIC, MIC, and 2 × MIC values) at 30 °C for 24 h. Following incubation, planktoic cells were aspirated with PBS, and then the biofilm was stained with crystal violet solution (0.1%, 200 μL) for 15 min. After that, the wells were washed twice with PBS, followed by ethanol (95%, 200 μL). In this assay, growth control and 1% DMSO (negative control) were also included. The biofilm formation quantification was done at OD_470nm_ using an iMark microplate reader (Bio-Rad Laboratories, Arvine, CA, USA). 

### 2.11. Confocal Laser Scanning Microscopy (CLSM)

In a parallel study, a CLSM study was performed to further establish the inhibitory effect of the biogenic ZnO NPs against *C. violaceum* biofilms [52]. Briefly, *C. violaceum* cells were grown on glass coverslips in 6-well cell culture plates under biofilm-forming conditions (30 °C, 24 h, and stationary growth) either with or without ZnO NPs (0.5 × MIC, MIC, and 2 × MIC). Afterwards, the planktonic cells were removed with PBS, and the biofilm was stained using FITC-ConA for 15 min. After a short incubation, cells were gently washed twice with sterile PBS to remove any excess stains, and subsequently analyzed at an excitation = 488 nm and emission = 520 nm, using a Zeiss Laser Scanning Confocal Microscope −780 and Airyscan (Carl Zeiss). 

### 2.12. Gene Expression

To study the consequences of ZnO NPs on the quorum-sensing marker genes (*cviL*, *vioA*, *vioB*, *vioD,* and *vioE*), RT-qPCR was done in treated and untreated *C. violaceum* cells, as described previously [34]. *C. violaceum* cells were exposed to MIC values of the biogenic ZnO NPs for three hours at 30 °C, followed by total RNA extraction usiεεng a High Pure RNA Isolation Kit according to the instructions provided by the manufacturer. By using a Nanodrop 2000 spectrophotometer, the RNA concentration was estimated. After that, cDNA was synthesized with the help of a RevertAid^TM^ cDNA Synthesis Kit, followed by RT-qPCR using different primers (Supplementary Materials Table S1). Crossing-point and melting curve analyses were used to determine the relative mRNA concentrations and the specificity of amplicons, respectively. The fold difference was calculated using the formula:

2^−(ΔΔCt)^ (2)

where ΔCt = mean Ct value of the target gene—the mean of housekeeping genes and ΔΔCt = ΔCt of the tested cells—ΔCt of the control cells.

### 2.13. Statistics

All the experiments were performed in triplicates, and the results were analyzed using two-way ANOVA and Dunnett’s multiple comparisons test. The data is represented as mean ± standard deviation, and *p* value < 0.0001 (*** *p*), <0.001 (** *p*) and <0.01 (* *p*) was taken as statistically significant.

## 3. Results and Discussion

**Figure 1:**
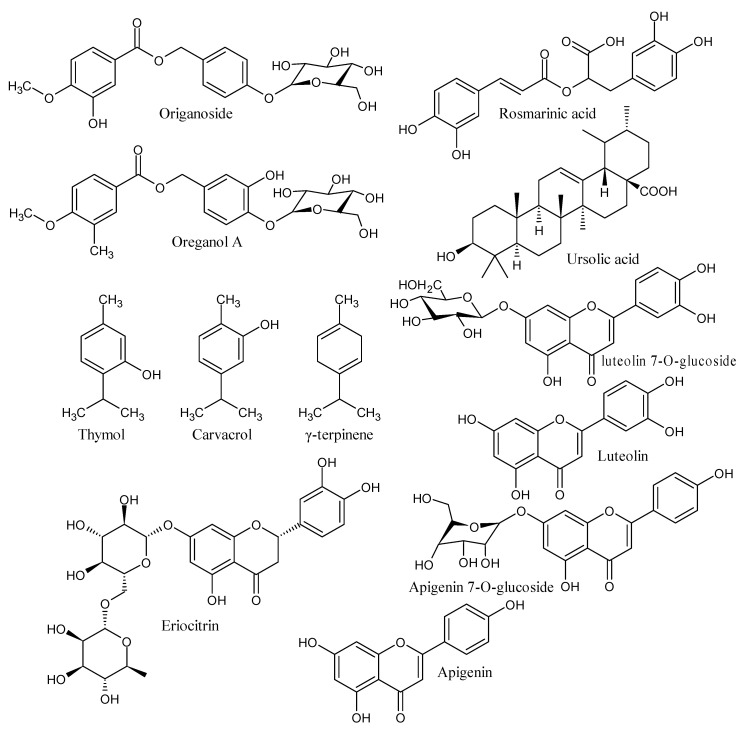
Chemical constituents present in the *Origanum vulgare extract*.

Primitive communities have employed plants for therapeutic and psychological healing characteristics since ancient times. In recent years, there has been a growing interest in the biological features of medicinal plants, the identification and evaluation of their therapeutic potential, and the identification of critical bioactive chemicals and prospective synergisms [53]. The current research on green nanomaterial fabrication using plant extracts has ushered in a new age [54]. For various purposes, many teams have reported the production of metal nanoparticles using aqueous extracts of plants [55]. The current research focuses on the production of ZnO NPs from *Origanum vulgare* aqueous leaf extracts. Because of its high volatile oil content, *Origanum vulgare* has long been used in folk medicine to treat diseases like asthma, bronchitis, coughs, diarrhea, indigestion, stomachache, menstruation disorders, general infections, inflammation-related illnesses, and diabetes [56,57]. The antioxidant activity of *Origanum vulgare* is due to phenolic compounds, such as flavonoids and phenolic acids, which are another type of abundant ingredient [58,59,60]. Furthermore, these phenolic antioxidants have various biological actions, including anti-ulcer, anti-inflammatory, antidiabetic, antiviral, cytotoxic, and antitumor properties, and they are responsible for *Origanum vulgare’s* health effects [61]. Figure 1 depicts the most frequent chemical compounds found in *Origanum vulgare* extract [62,63,64].

**Figure 2:**
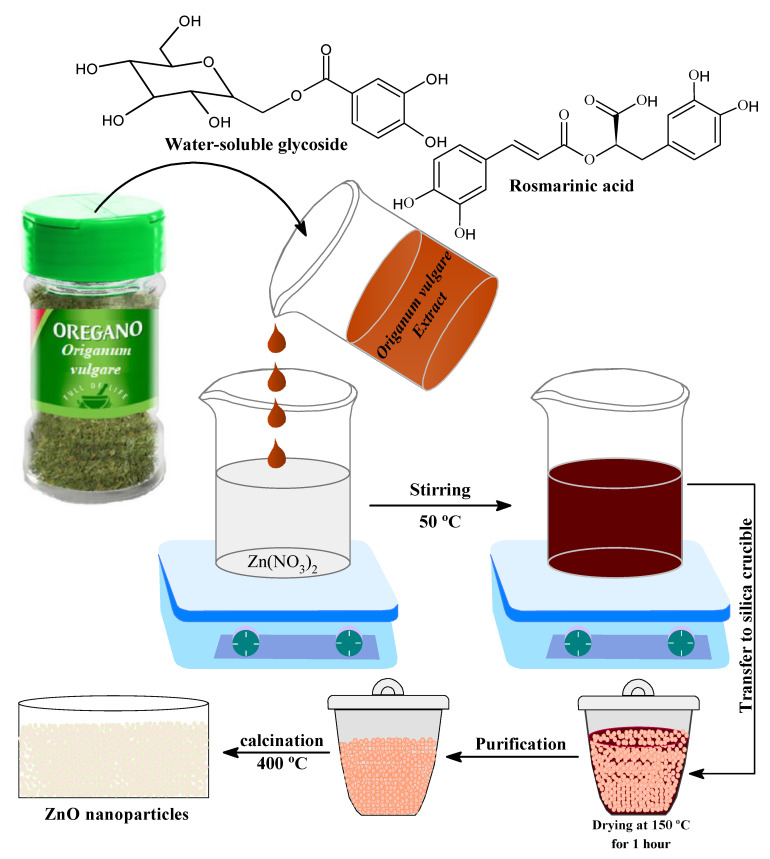
Reaction scheme for the synthesis of ZnO NPS.

Plant extracts are used as a replacement for stabilizing and reducing agents in the presence of several major bio components, such as terpenoids, alkaloids, phenolic agents, tannins, proteins, amino acids, polysaccharides, enzymes, vitamins, and saponins. This study presents a simple, green, and low-cost method for making stable and pure ZnO NPs using *Origanum vulgare* extract, free of chemical additives, such as bases, acids, and organic solvents, which are commonly used in conventional chemical methods. Figure 2 shows a schematic representation of the ZnO NPs green synthesis process. The current study was validated by performing spectroscopic and microscopic characterizations of prepared ZnO nanoparticles. 

### 3.1. Structural Characterization

**Figure 3:**
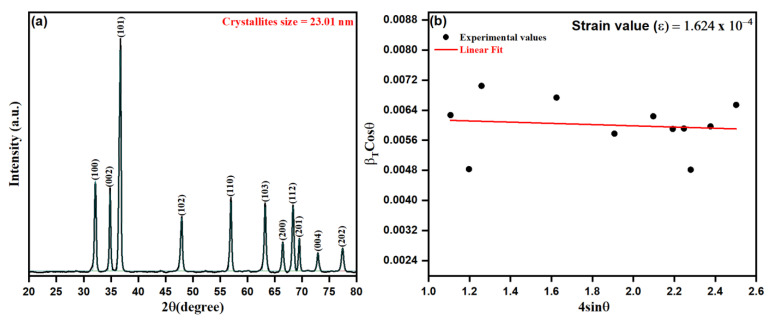
(**a**) XRD patterns of ZnO NPs, (**b**) Williamson–Hall plot for the ZnO NPs XRD pattern.

We adopted a green biosynthetic approach for developing ZnO NPs from *Origanum vulgare* extract during this study and, as such, the approach was characterized by microscopic and spectroscopic methods. Nanoparticles, in general, possess some basic physio-chemical properties, such as the surface-area-to-volume ratio, size, composition, solubility, stability, and purity, protruding their applicability and interactions in numerous fields [65]. Initially, the XRD technique was used to detect the structure of the crystalline lattice and calculate the crystalline size of particles, observed by the width (broadening) of the X-ray peaks. The as-prepared ZnO NPs from aqueous leaf extract of *Origanum vulgare* were analyzed for their crystalline structure by XRD spectrum as depicted in Figure 3a,b. The close perusal of Figure 3a shows that biogenic ZnO NPs possess 2θ intensity peaks at 32.13°, 34.82°, 36.67°, 47.91°, 56.95°, 63.22°, 64.46°, 68.34°, 69.48°, 72.90° and 77.41°, with the orientation plans (100), (002), (101), (102), (110), (103), (200), (112), (201), (004) and (202) corresponding to the hexagonal phase with a wurtzite structure as per file no. 36–1451 of the joint committee on powder diffraction standards (JCPDS) [66]. However, it is worth mentioning that the crystalline size as obtained by the Scherer equation from respective peak intensities shows varying sizes of crystallites from 19.67 to 28.78 nm. The dependence of the Scherer equation is mainly the contribution from the size reduction of crystallites and the instrumental factors. At the same time, it ignores the microstrain contribution leading to the peak broadening in the XRD pattern, where the Microstrain, being the short-range lattice strain arising from the point defects of crystallites, are not included in estimating the crystallite size using the Scherer equation [67,68]:

(3) D=0.9λ/βcosθ

where “*λ*” is the X-ray wavelength, *β* is FWHM (full width at half maximum) in radians, and *θ* is the diffraction angles. Relying on the above equation, the average crystalline size of biogenic ZnO NPs was estimated as 23.01 nm.

The microstrain contribution was evaluated in biogenic ZnO NPs as calculated upon Williamson–Hall XRD data interpretation and analysis, as depicted in Figure 3b. Thus, by the uniform deformation stress model [69,70], the contribution of microstain beside crystallite size could be reduced as per the equation:

(4) $$\frac{\beta \cos\theta }{\lambda }=\frac{k}{D}4\varepsilon \frac{\sin\theta }{\lambda }$$

where *ε* represents the microstrain contribution, *k* corresponds to the shape factor, *β* represents the instrumentally corrected full width at half maximum (FWHM) for a major peak under consideration, *λ* represents the X-ray wavelength (1.5406 Å), and D represents the adequate crystallite size [71]. In addition, micro-strain (*ε*) and the dislocation density (δ nm^−2^) were calculated by using the following equations:

(5) $$\varepsilon =\frac{\beta }{4\tan\theta }$$

(6) $$\delta =\frac{1}{D^{2}}$$

It is worth mentioning that the microstain contribution can be interpreted by a straight line of the slope ε against *k/D*, as shown in Figure 3b. The obtained diffraction data were utilized to estimate the related parameters, such as FWHM, crystallite size, and d-spacing, of biosynthesized ZnO NPs, as summarized in Table 1. In addition, the obtained average crystalline size of biogenic ZnO NPs from the intercept of the straight fit was calculated to be 21.26 nm. The obtained results of the positive slope *ε* (1.624 × 10^−4^) estimate the developed lattice strain in crystallites causing the additional broadening of peaks in the XRD pattern of biogenic ZnO NPs. It was observed that the average crystalline size from the Scherer equation was calculated to be lesser than the sufficient crystalline size by 23.40 nm. 

**Figure 4:**
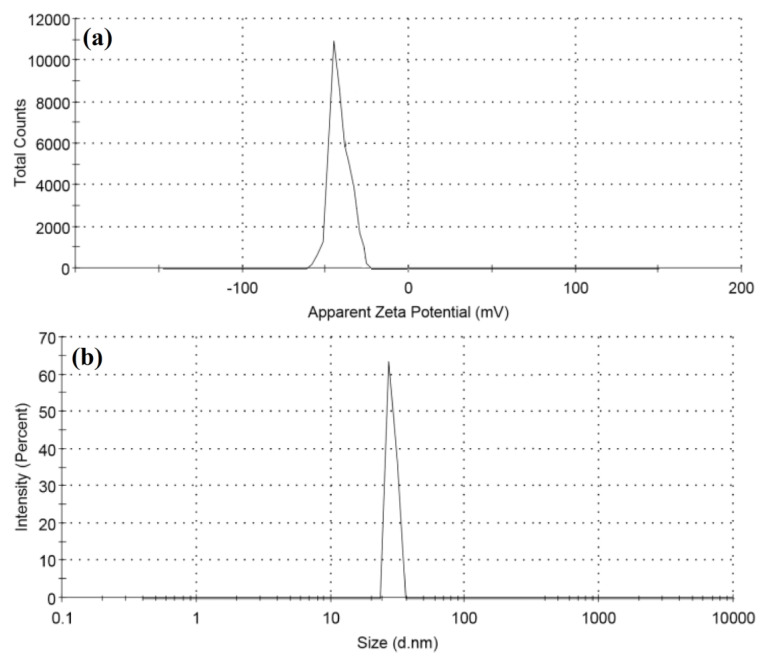
(**a**) Zeta potential and (**b**) particle size distribution of ZnO NPs.

The surface charge and stability of biosynthesized ZnO NPs were investigated using the zeta potential method. The zeta potential and size distribution of ZnO NPs are shown in Figure 4a,b, indicating that the nanoparticles are stable. The zeta potential of the ZnO NPs in distilled water was measured to be −14.7 mV, which is strongly anionic, indicating that the surfaces of biosynthesized ZnO NPs are coated with molecules that are largely involved in negatively charged groups, and which therefore indicates the stability of the nanoparticles [72,73]. The dispersion capacity of the biosynthesized ZnO-NPs is therefore verified and supported by the zeta potential measurements [74]. The negative surface charge is due to the extracted phytochemicals’ binding affinity with the NPs, which confers stability to the zinc oxide nanoparticles and reduces their aggregation potential [75]. As shown in Figure 4b, the hydrodynamic size of the particles was evaluated using dynamic light scattering and found to be 30.15 nm for the as-prepared ZnO NPs. When compared to the particle size acquired from SEM observations, the size distribution graph demonstrates that the particle size is bigger. The bias of the technology toward the measurement of bigger particles or even aggregates explains the increased size of the ZnO NPs measured by DLS [74]. The zeta potential of nanoparticles may be affected by the different functional groups of the biomolecules present in the plant extract adsorbed on their surface. These metabolites adsorbed on the surface of ZnO NPs and the zeta potential have a close association [76].

### 3.2. Morphology of ZnO NPs

**Figure 5:**
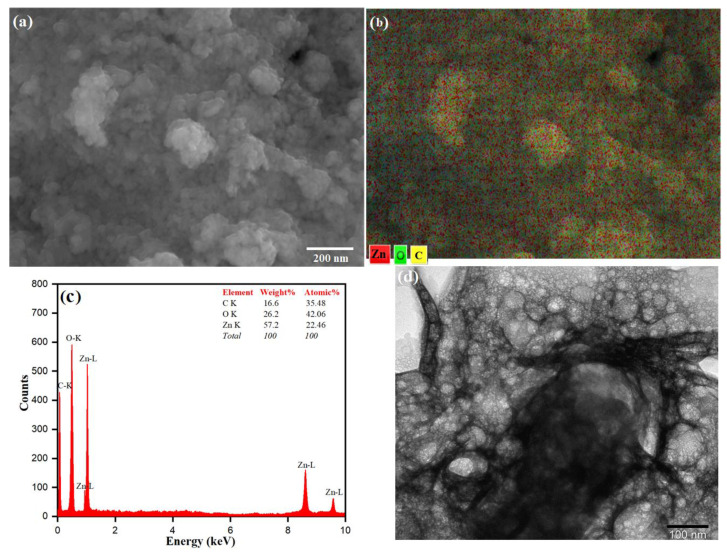
(**a**) SEM image, (**b**) Elemental mapping, (**c**) EDX spectrum and (**d**) TEM image of ZnO NPs.

The surface morphology of the as-prepared ZnO NPs was studied by SEM analysis as shown in Figure 5a. The SEM micrographs show that the particles are nearly spherical in shape, with particle sizes ranging from 20 to 25 nm. The SEM images also indicate that the ZnO nanoparticles show large-scale agglomeration owing to the presence of more capping/stabilizing agents on the surface of the ZnO NPs [77,78]. The presence of phytochemicals that play an essential role in fabricating and stabilizing biosynthesized ZnO NPs from *Origanum vulgare* extract influences the morphology and structure. The agglomeration is due to the polarity and electrostatic attraction of ZnO nanoparticles [79]. The particle size of the synthesized ZnO NPs was in close agreement with the findings of previously reported work [80,81]. The elemental mapping is observed in different colors for Zn (red), O (green) and C (yellow). These are shown in Figure 5b and in ZnO NPs, Zn and O are present in a higher density when compared to C. The elemental analysis of the prepared ZnO NPs was carried out by the energy-dispersive X-ray spectroscopy attached with the SEM (Figure 5c). The composition of each element contained in the as-prepared material is obtained from the EDX, which gives strong peaks of 57.2% for Zinc at 1.0, 8.6 and 9.5 eV and 26.2% for oxygen at 0.5 eV (Figure 5b), which are in good agreement with the previously reported work on the synthesis of ZnO NPs [82,83,84]. The presence of the carbon peak could be because of some carbon deposition during the calcination of the ZnO sample. The presence of carbon and oxygen also indicates stabilization of the as-prepared nanoparticles by phytochemicals present in the aqueous extract of *Origanum vulgare* [85,86]. The TEM analysis was used to understand the surface morphology and the size of the as-prepared ZnO NPs as shown in Figure 5d. The TEM image also reveals that the ZnO NPs agglomerate in a nearly spherical shape with an average particle size of 20–30 nm [87]. In the TEM image, a less intense layering is seen at the periphery of the nanoparticles, which could relate to the capping of biomolecules present in the aqueous extract of Origanum vulgare onto the resulting nanoparticles, indicating the role of biomolecules as reduction and capping agents [88]. It was discovered that the ZnO-NPs usually tended to agglomerate owing to high surface energy, which occurs when synthesis is performed in an aqueous solution, and potentially owing to densification, which results in a confined gap between particles [89]. Moghrovyan et al. reported that the phytochemical composition of *Origanum vulgare* extract includes water-soluble flavonoids, terpenes and polyphenolic compounds, which, being antioxidants, act as a reducing agent and are efficiently adsorbed onto the surface of NPs, thereby enhancing their biosynthesis and stability [90].

### 3.3. UV-Vis Spectroscopy of ZnO-NPs

**Figure 6:**
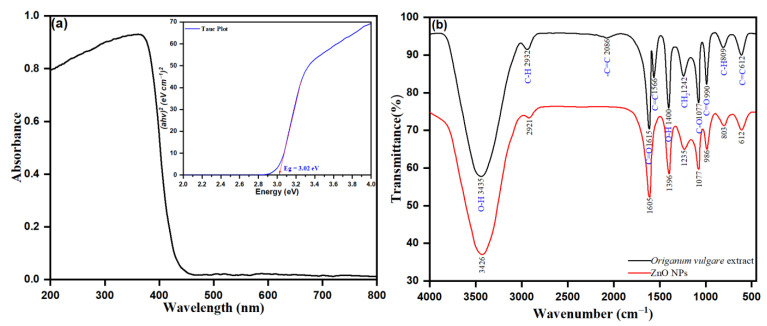
(**a**) UV-Vis absorption spectrum (inset shows corresponding Tauc plot) and (**b**) FTIR spectrum of ZnO NPs.

The spectroscopic analysis of the UV-Vis spectrum was examined to determine the role of phytochemicals, such as flavonoids, polyphenols, and terpenes, as bio-reductive agents of the biosynthesis of ZnO NPs from *Origanum vulgare* leaf extract. With the as prepared ZnO NPs in a biosynthetic approach, zinc nitrate is a precursor and *Origanum vulgare* leaf extract acts as the bio-reducing agents. Besides, in the reaction mixture, the phytochemicals are responsible for bio-reducing Zn^2+^ to Zn^0^ and are determined by UV-Vis diffuse reflectance spectroscopy (DRS). The UV-Vis diffuse reflectance spectroscopy (DRS) was used to investigate the optical properties of the synthesized NPs within a wavelength of 200 to 800 nm at room temperature. The prepared pellets (pressure of 300 MPa) within the dimensions 6 mm × 1 mm containing the powdered ZnO NPs are shown in Figure 6a. The UV-visible broad absorption peak intensity observed at *λ*_max_ of 460 nm was from the intensive band-gap absorption, behind the electron transfer of the valance band (Ev) to the conduction band (Ec) of orbitals O2p → Zn3d in ZnO NPs. The precursor zinc nitrate solution without a reduction agent possesses no surface plasmon resonance (SPR) band. However, the presence of *Origanum vulgare* leaf extract as a bio-reducing agent upon reduction of Zn^2+^ to ZnO nanoparticles was accentuated by visible peak intensities. The presence of a peak intensity at *λ*_max_ of 460 nm confirmed the bioreduction of Zn^2+^ towards the ZnO nanoparticles. The observed hyperchromic shift towards a higher wavelength, the redshift of the peak intensity at *λ*_max_ of 335 to the *λ*_max_ of 460 corresponding to the leaf extract, indicates the role of phytochemicals in the leaf extract as a bio-reducing agent. In addition, the observed strong adsorption peak of biogenic ZnO NPs in the UV-vis region indicates the applicability of ZnO NPs for photocatalytic action and for medicinal uses, such as in ointments and sunscreen creams, and as an antiseptic [91]. However, the UV-vis diffuse reflectance spectroscopy was applied to calculate the bandgap energy by using the following equation:

(7) $$\alpha =\frac{c{\left(hv-E_{Bulk}\right)}^{1/2}}{hv}$$

where α represents the absorption coefficient (*α*= 2.303 A t^−1^), A represents the absorbance, t represents the thickness of the ZnO sample sheet, c represents the constant, *h* is the planks constant, *ν* represents the photon energy vibration frequency, and E*_bulk_* represents the bulk ‘band gap’. The curve was plotted using (*αhν*)^2^ as a function of the photon energy for the photon energy (*h*), and the direct bandgap was discovered to be 3.02 eV by inferring the linear component of the graph, as shown in Figure 6a.

### 3.4. FTIR Analysis of Biogenic ZnO NPs

FTIR spectroscopy was a valuable technique in determining the functional groups in *Origanum vulgare* extract involved in the biosynthesis of ZnO NPs. In general, the FTIR analysis demonstrates the reduction and the appearance of new possible functional groups, advanced upon formation of new bonds during biosynthesis through reduction, fabrication, or capping/stabilization of NPs. In our study, a close perusal of Figure 6b shows the spectrum of pure ZnO (red colored line) and the obtained *Origanum vulgare* extract (black colored line). The spectra of pure ZnO NPs in Figure 6b retained the intensity peak of 3426, 2921, 1605, 1396, 1236, 1077, 986, 803, and 612 cm^−1^, respectively. The peak intensity at 3426 cm^−1^ was observed to be –OH stretching of phenolic alcohol, with 2921 cm^−1^ corresponding to C–H stretching of alkane, and 1605 cm^−1^ attributed to the presence of -C=O stretching vibration due to carboxylic acids and esters. The peaks at 1396 and 1236 cm^−1^ were observed after –OH bending vibration modes of phenolic groups and –CH_2_ stretching vibrations of carbon chains, respectively. The characteristic peak at 1077 cm^−1^ corresponds to the C–O–C stretching vibrations. The peak at 803 cm^−1^ after the corresponding C–H out-of-plane bending modes in aromatic phenolic compounds and the peak intensity at 612 cm^−1^ determined after the characteristic bond singles of Zn-O confirmed the synthesis of ZnO NPs [92]. 

The peaks within the range of pure ZnO NPs were observed in *Origanum vulgare* extract (black colored line), as shown in Figure 6b. As such, the observed intensity peaks at 3435, 2932, 1615, 1242, 1077, 890, and 612 cm^−1^ were within the range observed in pure ZnO NPs. Though the peaks observed from *Origanum vulgare* extract (black color line), those at 3435 cm^−1^ are attributed to the presence of O–H and N–H stretching vibration of proteins, amides, and phenols in the biosynthesized ZnO NPs. The corresponding C–H stretching of alkenes was observed at 2932 cm^−1^. Peak intensities at 2086, 1566 and 1615 cm^−1^ were ascribed to C=C/N–H/C=O stretching vibrations due to alkenes, amides, carboxylic acids, and esters. The observed peaks at 1242 (–OH bending vibration modes of phenolic groups), 1077 (C–O–C stretching vibrations), 890 (C–H out of plane bending modes in aromatic phenolic compounds), and 612 cm^−1^ (bond singles of Zn–O), respectively, emphasized the successful biosynthesis of ZnO NPs during this study. Overall, the results of FTIR confirmed the successful biosynthesis of ZnO NPs and demonstrated that the role of phytochemicals of *Origanum vulgare* leaf extract as reducing/stabilizing agents on the surface of the ZnO-NPs is well established.

### 3.5. Probable Biosynthetic Mechanism of ZnO NPs upon Fabrication in Origanum vulgare Extract

**Figure 7:**
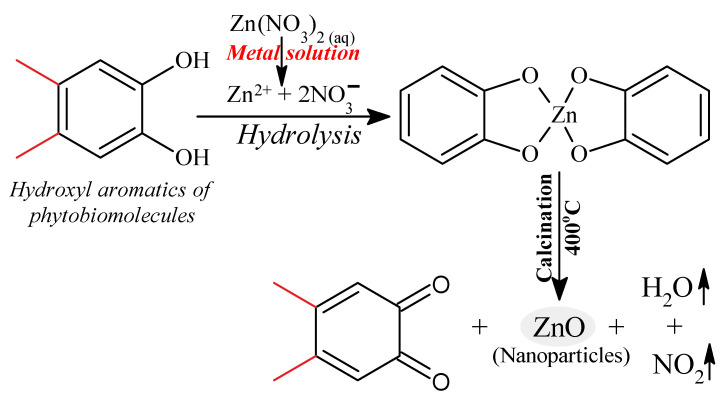
The chemical mechanism of ZnO nanoparticle formation.

In general, the green synthetic approach of ZnO NPs involves utilizing a phytochemical agent viz. polyphenols (quercetin), to reduce Zn^2+^ to ZnO NPs. Figure 7 shows a proposed reaction pathway to produce ZnO NPs from *Origanum vulgare* extract, in which a ligation process occurs between the functional components of the phytomolecules in the *Origanum vulgare* extract and the zinc precursor. *Origanum vulgare* extract contains organic compounds (flavonoid, polyphenolic compounds) that serve as binding agents. One of the extract’s components, the hydroxyl aromatic ring groups of the biomolecules, forms complex ligands with zinc ions. As a result, nanoparticles are stabilized and created by the nucleation and shaping process. In the reaction medium, zinc nitrate provides Zn^2+^ ions, which are reduced by the presence of phytochemicals of the aqueous extract of *Origanum vulgare*, mainly quercetin (a polyphenol), to produce ZnO, as depicted in Figure 7. When an organic solution is calcined at 400 degrees Celsius, it decomposes, releasing ZnO NPs [93].

### 3.6. TGA-DTG Analysis of Biogenic ZnO NPs

**Figure 8:**
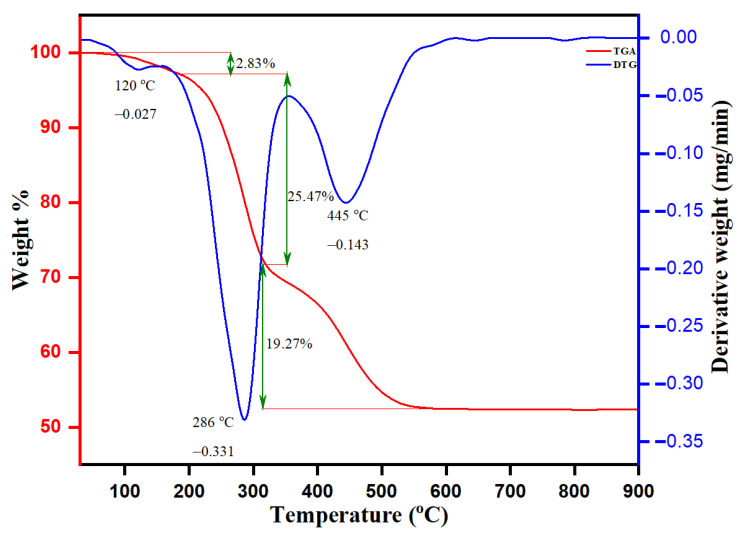
TGA-DTG analysis curve of ZnO NPs.

The thermal stability and degradation pattern of biogenic ZnO NPs were established by TGA and DTG analysis, as depicted in Figure 8. The thermal degradation (TGA) was stable up to 750 °C, with an approximate weight loss of about 47.57%. The weight loss was observed in ranges between 50–250, 250–310, 310–350 and 350–750 °C with percentage weight losses of 2.83%, 19.27% and 25.47%, respectively. The observed weight loss by 2.83% was in the TGA range between 50 and 250 °C and another observed DTG intensity peak at 120 °C with a derivate weight rate at 0.027 mg/min. The intensity DTG peak at 286 °C with the derivative rate at 0.331 mg/min was attained by the evaporation of moisture adsorbed onto the surface of biogenic ZnO NPs and thermal degradation of volatile phyto-compounds. The observed sharp peaks of DTG at 445 °C with a derivative weight rate at 0.143 mg/min were after the decomposition of protein, carbohydrates, flavonoids, and phenolic acids of leaf *Origanum vulgare* extract responsible for the stabilization of biogenic ZnO NPs. The transition change observed in the temperature range between 250 and 310 °C of TGA was ascribed to the presence of the ongoing endo and exothermic processes. In a mixture sample, such an endothermic reaction leads to the thermal decomposition of organic compounds bound onto the surface of ZnO NPs with a weight loss of 19.27%. In addition, the TGA analysis pronounces that the weight loss of 25.47% (between 310 and 350 °C) upon another simultaneous transition was due to thermal degradation of the adsorbed aromatic organic compounds on the surface of biogenic ZnO NPs. After 550 °C, the straight line shows that complete thermal decomposition of the sample with phytochemical composition on the surface of biogenic ZnO NPs occurred, serving as stabilizing or reducing agents. 

### 3.7. Antimicrobial Susceptibility Testing

Antimicrobial susceptibility testing of ZnO NPs was confirmed by determining the MIC and MBC against the bioreporter strain *C.* *violaceum*. The MIC values demonstrated that ZnO NPs inhibit the growth and survival of *C. violaceum* at 4 µg/mL. The MBC results confirmed that ZnO NPs have bactericidal activity, with an MBC value of 16 µg/mL (Table 2). However, the positive control (ampicillin) showed MIC and MBC values of 2 and 4 µg/mL, respectively. Furthermore, at a sub-inhibitory concentration of ZnO NPs (1 µg/mL), cell growth without violet color was observed, recorded as MQSIC. 

The increasing use and abuse of antibiotics and biofilms, and several other factors, such as antibiotic resistance, have drastically increased over the past few decades. Therefore, despite introducing several new antibacterial drugs, treating drug-resistant pathogens in clinics has become challenging. Therefore, besides developing new antibiotics, it has become crucial to use novel strategies in developing antibacterial drugs. Biofilm formation and quorum sensing have gained much attention as emerging drug targets [94].

ZnO NPs were synthesized in the quest to discover novel nanoparticles with antibacterial and antiquorum-sensing properties. The antimicrobial susceptibility results show that ZnO NPs have potent antibacterial activity against the planktonic form of *C. violaceum* at higher concentrations. The obtained results are congruent with the literature, where different types of nanoparticles possess antimicrobial activities [95,96,97,98]. 

Furthermore, at sub-inhibitory concentrations, these NPs could also suppress quorum sensing in *C. violaceum* cells, whereas, at these low values, the ZnO NPs have no inhibitory effect on bacterial growth. Therefore, the inhibition of quorum sensing is not related to bacterial growth inhibition or cell death. Our results are related to previous findings, where different nanoparticles possess antiquorum-sensing activity against various bacterial pathogens [99,100]. In a separate study, selenium- and tellurium-based nanoparticles inhibit bacterial growth at higher concentrations. In contrast, these NPs interfere with bacterial quorum sensing and biofilm formations [46].

### 3.8. Antiquorum-Sensing Property

**Figure 9:**
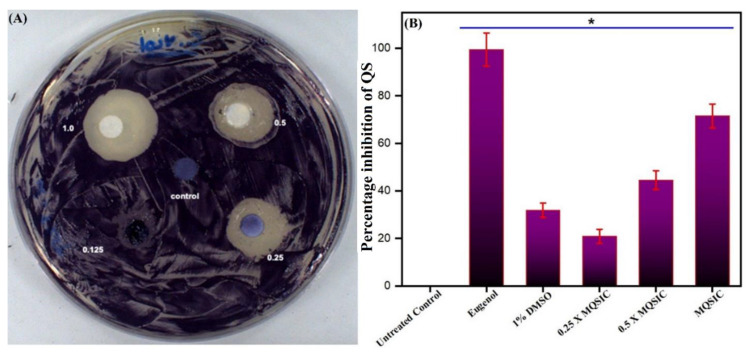
(**A**). Disc diffusion assay. The plate represents the antiquorum-sensing property of ZnO NPs at MQSIC and sub-MQSIC values. (**B**). Quantitative inhibition of violacein production by ZnO NPs. *C. violaceum* cells were treated with MQSIC and sub-MQSIC values of the ZnO NPs. The bar indicates healthy cells with no inhibition in violacein production, while eugenol and 1% DMSO represent positive and negative controls, respectively. Data represents means ± SD of three different values; * *p* ≤ 0.01.

Qualitative: A qualitative assay for the antiquorum-sensing property of the ZnO NPs revealed their antiquorum properties (Table 2). The disc diffusion assay resulted in clear and turbid zones against the violet background and demonstrated the suppression of bacterial growth and the antiquorum property of the ZnO NPs, respectively (Figure 9A). The maximum antiquorum-sensing activity imparted by the ZnO NPs was represented by the formation of a 19-mm (average diameter) opaque zone against the violet background (Table 2).

Further, validation and quantification of the antiquorum-sensing property of the biogenic ZnO NPs was done spectrophotometrically by estimating the production of violacein pigment in treated and untreated *C. violaceum* cells. The results show that ZnO NPs significantly blocked the synthesis of violacein pigment at 1 µg/mL (Table 2). In addition, the antiquorum-sensing activity was found to be dose dependent, with low quorum-sensing inhibition at sub-quorum-sensing inhibitory concentrations and higher inhibition at quorum-sensing inhibitory concentrations (Figure 9B). A significant reduction in violacein pigment production was measured at sub-quorum-sensing and quorum-sensing inhibitory concentrations. In contrast, there was no change in the numbers of CFU/mL, suggesting that the pigment production was inhibited due to the inhibition of quorum sensing and was not due to hampered bacterial growth. The results obtained in the DMSO controls were similar to those from untreated cells, with more than 80% pigment production, denoting that DMSO (1%) does not affect the growth and survival of cells and causes minimum inhibition of violacein production. 

Quorum sensing, a cell-to-cell interaction that allows unicellular microorganisms to behave as multicellular organisms, is a controlled process that allows numerous bacterial functions [101]. Previous studies have also indicated that quorum sensing in *C. violaceum* is vital for developing pathogenesis in this bacterium. Therefore, antiquorum-sensing compounds can decrease the bacterium’s pathogenicity and possibly that of many other bacterial species [102]. Furthermore, potential antiquorum-sensing compounds are known to mimic the structure of autoinducers [103]. We hypothesize that ZnO NPs also interfere with the autoinducers and thereby disturb the communication mechanisms of this bacterium. Despite numerous studies reporting antibacterial and other therapeutic applications of nanoparticles, there is a clear gap in applying nanoparticles as antiquorum-sensing agents. 

### 3.9. Biofilm Inhibition Activity

**Figure 10:**
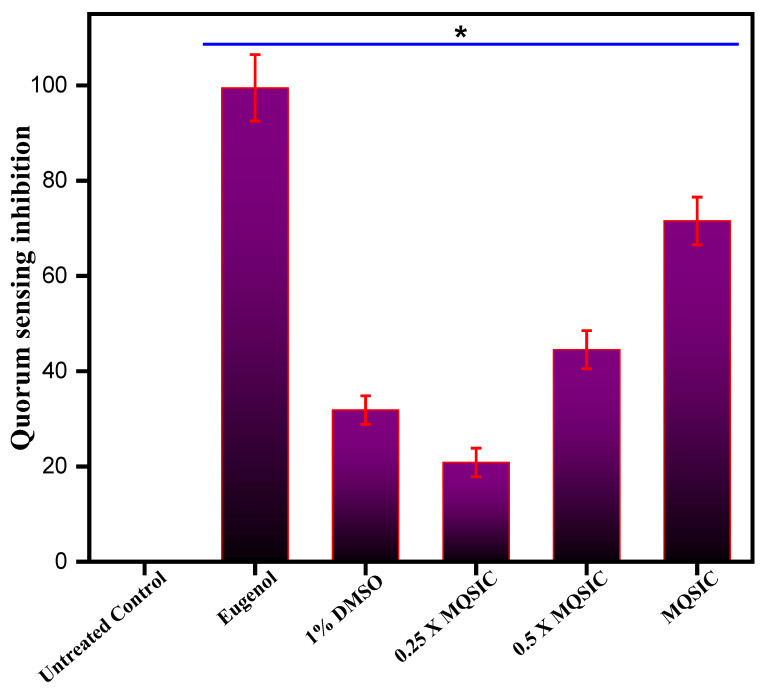
Percentage of biofilm formation in treated and untreated *C. violaceum*. The percentage of biofilm formation was determined by staining treated and untreated *C. violaceum* cells with 0.1% crystal violet solution. The control bar indicates untreated cells, accepted as 100% biofilm-forming cells. Data represents means ± SD of three different values; * *p* ≤ 0.01.

Crystal violet staining: The effect of ZnO NPs on biofilm formation was determined by crystal violet staining. The capability of biofilm formation by *C. violaceum* was altered after being treated with ZnO NPs at different concentrations (Figure 10). The results demonstrated a marked inhibition in biofilm formation, and the impact was found to be dose dependent. In conclusion, treatment with ZnO NPs resulted in a 24–78% inhibition in biofilm formation, and maximum inhibition was recorded at a concentration of 2 × MIC. 

### 3.10. Confocal Laser Scanning Microscopy (CLSM) 

**Figure 11:**
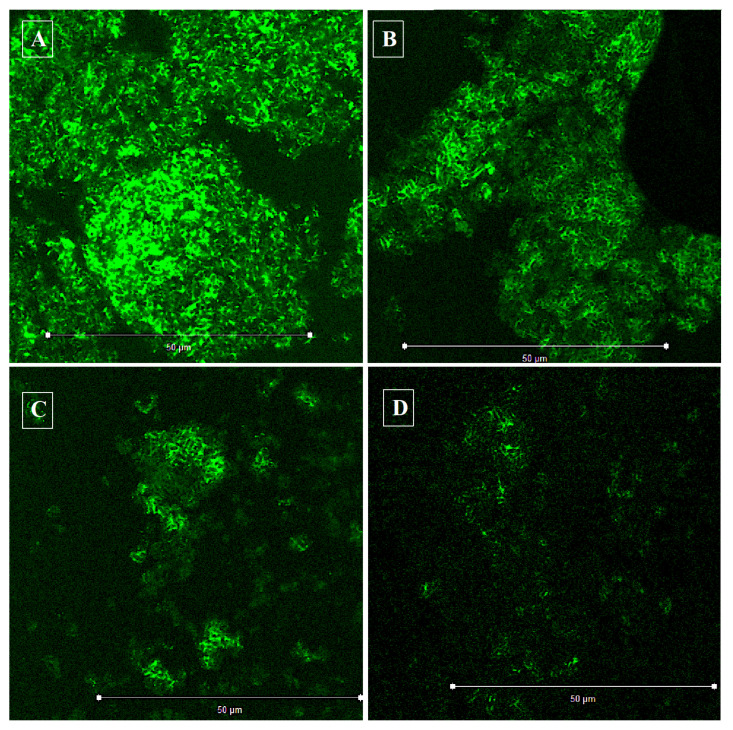
Confocal images of *C. violaceum* biofilms. The figure represents the efficacy of the biogenic ZnO NPs in preventing biofilm formation in *C. violaceum*. (**A**) Untreated control (**B**) 0.5 × MIC of ZnO NPs (**C**) MIC of ZnO NPs (**D**) 2 × MIC of ZnO NPs.

The CLSM analysis validated the efficacy of the biogenic ZnO NPs against *C. violaceum* biofilm development. It was observed that biogenic ZnO NPs at an inhibitory and sub-inhibitory concentration negatively regulated the biofilm formation in *C. violaceum* (Figure 11). Specifically, at 8 μg/mL (2 × MIC), the biogenic ZnO NPs showed significantly reduced biofilm formation. However, the biofilm inhibition was found to be concentration dependent.

Biofilm formation has caused great worry and concern in clinical settings and has been directly linked to drug resistance [104]. The biofilm inhibition assay results were consistent with CLSM studies, revealing that biogenic ZnO NPs show drastically reduced biofilm formation in *C. violaceum.* As is evident from the results, it is crucial to understand that biofilm formation reduction is not linked to bacterial killing, as significant growth was observed in the microtiter plate wells after exposure to the nanoparticles. Our results agree with the aforementioned findings, where lower natural product concentrations and their derivatives prevent biofilm formation [105]. Furthermore, different types of nanoparticles inhibit biofilm formation in various bacterial species. For example, in a study by Mohanta and co-workers, newly synthesized silver nanoparticles were reported to possess antibacterial and anti-biofilm activities against *Pseudomonas aeruginosa*, *Escherichia coli*, and *Staphylococcus aureus* [106]. Previous studies have also reported that mesoporous calcium-silicate nanoparticles possess antibacterial activity against *Enterococcus faecalis* biofilms in different degrees [107]. 

In addition, the present results revealed the mechanism of the antibacterial action of ZnO NPs, which could be linked to the inhibition of biofilm formation and the prevention of quorum sensing within the cells. On the other hand, biofilms have also been established as problematic because they enable bacteria to survive hostile environments and antibiotics [108,109]. Therefore, biofilms amplify the challenge in using conventional antibiotics to suppress the infection altogether [110]. This increases the demand for new antibacterial drugs, which can either inhibit biofilm formation or penetrate biofilms to reach the pathogens. Biofilm formation has also been directly controlled by quorum sensing. Therefore, quorum sensing is a valid and completely proven strategy against bacterial infections. It will stop them from communicating and developing biofilms, which are their defensive armor against antibiotics [105,111]. Further studies using different bacterial pathogens will unveil several factors related to these ZnO NPs’ antiquorum-sensing and antibiofilm activities. 

### 3.11. Effect on Quorum Sensing-Associated Marker Genes

**Figure 12:**
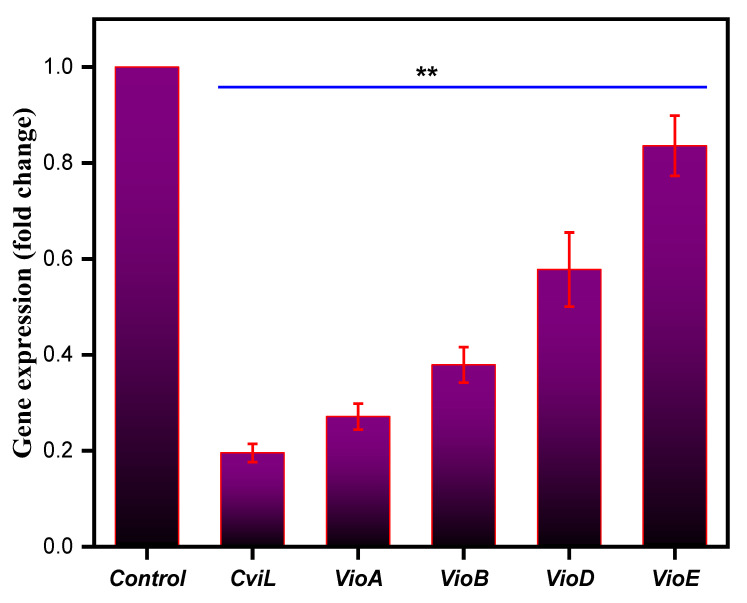
Relative expression of genes controlling quorum sensing in *C. violaceum* when treated with MIC values of the ZnO NPs. The gene expression was normalized to that of the housekeeping genes, and the expression is compared to that of untreated cells. Data represents means ± SD of three different values; ** *p* ≤ 0.001.

To further confirm the antiquorum-sensing potential of ZnO NPs at the gene level, the ability of ZnO NPs to alter the expression of quorum-sensing marker genes in *C. violaceum*, such as *cviL*, *vioA*, *vioB*, *vioD* and *vioE*, was determined. Compared to the untreated negative control, the NPs-treated *C. violaceum* cells showed a drastic decrease in the expression level of the marker genes responsible for quorum sensing (Figure 12). Furthermore, at the MIC value, ZnO NPs downregulated the expression of all marker genes by 1.2–4.1-fold, compared to that of untreated control cells, which were set at 1.0 (Figure 12). Thus, our results clearly show that ZnO NPs lowered the expression of the genes responsible for quorum sensing in *C. violaceum*, triggering inhibition of *N*-acyl-l-homoserine lactone synthase, and in response blocking quorum sensing in *C. violaceum.* These results also support earlier findings, where a putative repressor protein, VioS, could negatively regulate quorum-sensing genes in *C. violaceum* [112]. In another study, metal and metal oxide nanoparticles were responsible for quenching quorum sensing in Gram-negative bacteria by targeting the quorum-sensing genes [45]. Similarly, Singh and co-workers reported the potential of microfabricated silver nanoparticles as quorum-sensing agents by significantly downregulating quorum-sensing genes in *Pseudomonas aeruginosa* [113]. 

It will be interesting to compare these NPs with antibiotics, such as kanamycin, which stimulate virulence and biofilm formation in bacteria at lower concentrations by upregulating quorum-sensing genes [114]. In contrast to these findings, our results suggested that these ZnO NPs at sub-inhibitory concentrations target quorum sensing by downregulating the related genes. In total, the antiquorum-sensing effect of biogenic ZnO NPs was observed at the molecular level, which demonstrates that there is a significant impact on the expression of critical genes responsible for quorum sensing in *C. violaceum* cells. However, all these results need to be further verified by employing different bacterial strains, which will strengthen these claims further. 

## 4. Conclusions

In conclusion, this study targeted the synthesis of biogenic ZnO NPs, which displayed antibacterial, antiquorum-sensing, and antibiofilm properties. As a result, growth and biofilm formation were reduced at higher concentrations of these nanoparticles, whereas quorum-sensing activity was depleted at sub-inhibitory concentrations. Furthermore, the expression level of quorum-sensing genes was downregulated when treated with ZnO NPs in *C. violaceum*. Collectively, our results demonstrate that targeting quorum sensing and biofilm formation by biogenic ZnO NPs can be used as a novel idea in developing drugs that can counter cell-to-cell communication, prevent biofilm formation, and ultimately prevent infections and bypass drug resistance. However, further investigation with different bacteria, including sensitive and resistant pathogens, is required to strengthen the potency of biogenic ZnO NPs against quorum sensing and biofilm formation and future in vivo studies. 

## Figures and Tables

**Table 1:** XRD data and crystallite size of ZnO NPs.

Position (2θ°)	Crystallographic Planes (hkl)	d-Spacing (Å)	FWHM	Crystallite Size (d) (nm)
32.13	100	2.78338	0.37383	22.11
34.82	002	2.57389	0.29024	28.68
36.67	101	2.44856	0.42528	19.67
47.91	102	1.89688	0.42229	20.58
56.95	110	1.61550	0.37656	24.00
63.22	103	1.46946	0.41969	22.22
66.46	200	1.40563	0.40421	23.49
68.34	112	1.37135	0.40952	23.44
69.48	201	1.35163	0.33586	28.78
72.90	004	1.29644	0.42515	23.23
77.41	202	1.23178	0.48021	21.20

**Table 2:** Results displaying values of MIC, MBC, MQSIC, and zone of turbidity (ZoT) in mm for the biogenic ZnO NPs against *C.* *violaceum*.

Nanoparticles	MIC (μg/mL)	MBC (μg/mL)	MQSIC (μg/mL)	ZoT (MIC)
Biogenic ZnO NPs	4	16	1	19 ± 0.03
Ampicillin	2	4	Not tested	Not tested

## Data Availability

All relevant data are within the manuscript.

## Supplementary Materials

The following are available online at https://www.mdpi.com/article/10.3390/pharmaceutics13111743/s1. Table S1: List of primers employed for RT-qPCR experiment.
