# Supplementary material for: Biogenic ZnO Nanoparticles Synthesized from Origanum vulgare Abrogates Quorum Sensing and Biofilm Formation in Opportunistic Pathogen Chromobacterium violaceum
Source: Pharmaceutics. 2021 Oct 20;13(11):1743. doi: 10.3390/pharmaceutics13111743 (PMC8625425; doi:10.3390/pharmaceutics13111743)
Supplement: Supplementary file 1 [file pharmaceutics-13-01743-s001.zip › pharmaceutics-1382316-supplementary.pdf]

pharmaceutics

MDPI

Supplementary Material

Biogenic ZnO Nanoparticles Synthesized from *Origanum vulgare* Abrogates Quorum Sensing and Biofilm Formation in Opportunistic Pathogen *Chromobacterium violaceum*

Majid Rasool Kamli [1,2,*], Maqsood Ahmad Malik [3,*], Vartika Srivastava [4], Jamal S. M. Sabir [1,2], Ehab Hussain Matter [1] and Aijaz Ahmad [4,5]

1 Department of Biological Sciences, Faculty of Science, King Abdulaziz University, Jeddah 21589, Saudi Arabia; jsabir2622@gmail.com (J.S.M.S.); ematter@kau.edu.sa (E.H.M.)
2 Center of excellence in Bionanoscience Research, King Abdulaziz University, Jeddah 21589, Saudi Arabia
3 Department of Chemistry, Faculty of Science, King Abdulaziz University, Jeddah 21589, Saudi Arabia
4 Clinical Microbiology and Infectious Diseases, School of Pathology, Faculty of Health Sciences, University of the Witwatersrand, Johannesburg 2193, South Africa; vartika.srivastava@wits.ac.za (V.S.); Aijaz.Ahmad@wits.ac.za (A.A.)
5 Infection Control Unit, Charlotte Maxeke Johannesburg Academic Hospital, National Health Laboratory Service, Johannesburg 2193, South Africa
* Correspondence: mkamli@kau.edu.sa (M.R.K.); mamalik@kau.edu.sa (M.A.M.)

Table S1. List of primers employed for RT-qPCR experiment.

Gene	Forward Primer	Reverse Primer
cviL	5'-GGATCCCCGTAGGCAAAGAACTAA-3'	5'-GAATTCTTGTGTCTGAACGCCA-3'
vioA	5'-GGATCCGCCCAAAGCCAGACTA-3'	5'-GAATTCTGAACGGCACGATTGA-3'
vioB	5'-GGCCTATTACGAGCTGGTCT-3'	5'-TGGGCCAGGTATTTGAGGAA-3'
vioD	5'-GCCGCAACAAGTACATCTGG-3'	5'-AACACCTTGGCGACGTATTC-3'
vioE	5'-TGGAAGCAGAAGGTGGCC-3'	5'-GCGGCGTCCAGGTACAAC-3'
16S	5'-GCGCAACCCTTGTCCTTAGTT-3'	5'-TGTCACCGGCAGTCTCCTTAG-3'
